# Liver and Pancreas Transplantation

**Published:** 1985-04

**Authors:** 


### Liver and Pancreas Transplantation

This was the title of the 4th Marjorie Budd Lecture given by **Professor Roy Calne F.R.S.** on 11th January at the Jenner Centre, Bristol Royal Infirmary

Professor Calne performed the first liver transplant in this country in 1968 at Addenbrookes Hospital, Cambridge, shortly after he was appointed to the first Chair of Surgery in the University of Cambridge. He first described some of the experimental work carried out before work on humans could begin. He paid tribute to the pig which was able to take grafts from unrelated animals of its species and showed a slide of a pig 10 years after a successful transplant. He also paid tribute to the early experimental work done in Bristol by Joe Peacock and John Terblanche.

When a donor liver has been obtained 10 hours is the maximum delay before it can be successfully grafted into a patient and helicopters and police motor cycle teams are needed for its transport. No account of tissue type is taken as this would further prolong the delay. The most difficult part of the operation is often removing the diseased liver from the patient especially when there has been previous surgery. Most of the postoperative complications arise from the biliary connections. It is always necessary to cross match 30 units of blood and massive physiological changes are produced. Sometimes it is advisable to assist the circulation with a bypass taking blood from the inferior vena cava to the aorta without using heparin as this produces severe bleeding problems. Transplants are always orthotopic, ectopic transplants – placing the transplant in some other part of the abdomen has not proved successful.

The advent of Cyclosporin A has greatly simplified the problems of immune suppression. It is much better than Imuran and steroids are unnecessary. However, Cyclosporin is nephrotoxic and the search is now on for less harmful variants.

There is enormous public sympathy for the sick child and Esther Rantzen's programme on little Ben Hardwick had been very helpful in making the public aware of the need and use for donor organs and had gone some way to remedying the disaster of the Panorama programme on transplantation.

The postoperative care in these cases is so incredibly complex that there has to be a 1 : 1 doctor/patient ratio in the Intensive Care Unit.

They have now done 186 liver transplants at Cambridge, the longest survivor being 9 years. Out of 14 children grafted 12 have functioning grafts. Four of these children had to have a second transplant and two of these are functioning. A slide was shown of one of these cases in which the child was in coma and the operation done virtually without anaesthesia. The liver was necrotic and the vessels thrombosed. Another liver was put in and within hours the child was conscious a photograph a few months later showed a smiling healthy looking child. By far the largest number of liver transplants in the world has been done by Starzl in the U.S.A.; his survival rate at one year is now 60%. The Cambridge results are 70–80% one year survival for children and 50% for adults. We were shown a gallery of survivors including a District Nurse, a skier, a tennis teacher and a runner who runs 10 miles a day and was disqualified from the transplant Olympics because he had received a liver and not a kidney.

The second part of his talk was about transplantation of the pancreas. The best indication for such a transplant is a diabetic patient also suffering from renal failure who is in danger of losing his sight from retinitis. A kidney and about half a pancreas are transplanted at the same time. Various techniques have been used. Simple islet transplants in man have always failed. Three techniques for major transplants have been used. The first attempted to get rid of the exocrine secretion of the pancreas either by ligating the duct or by injecting it. A high failure rate led to a second technique of implanting the duct into a Roux loop constructed in the pelvis. This met with a fair degree of success but a better method, now under trial, seems to be the 'Paratopic' method in which the duct of the transplant is brought into the stomach and the vascular connections are made with the splenic vessels with a self-closing a-v shunt to assist patency. So far nine of these transplants have been done and seven have been successful.

Professor Calne's talk ended with a photograph of four young people sitting on the lawn outside the hospital who had had successful double grafts and from whom a death sentence or the threat of blindness had been removed, at any rate for the immediate future.

When one reflects on the skill, the ingenuity and the sheer determination needed to succeed in such a pioneering enterprise and the cost of it all in terms of man and woman hours, equipment and drugs, it does reconcile at least your reporter to the government's unpopular attempt to claw the cost of at least some of it out of the nation's drug bill.

